# Intraoperative assessment of skull base tumors using stimulated Raman scattering microscopy

**DOI:** 10.1038/s41598-019-56932-8

**Published:** 2019-12-31

**Authors:** Kseniya S. Shin, Andrew T. Francis, Andrew H. Hill, Mint Laohajaratsang, Patrick J. Cimino, Caitlin S. Latimer, Luis F. Gonzalez-Cuyar, Laligam N. Sekhar, Gordana Juric-Sekhar, Dan Fu

**Affiliations:** 1 Department of Chemistry, University of Washington, Seattle, 98125-1700 USA; 2 School of Medicine, University of Washington, Seattle, 98125-1700 USA; 3 Department of Pathology, University of Washington, Seattle, 98125-1700 USA; 4 Department of Neurological Surgery, University of Washington, Seattle, 98125-1700 USA

**Keywords:** Imaging and sensing, Cancer imaging, Surgical oncology

## Abstract

Intraoperative consultations, used to guide tumor resection, can present histopathological findings that are challenging to interpret due to artefacts from tissue cryosectioning and conventional staining. Stimulated Raman histology (SRH), a label-free imaging technique for unprocessed biospecimens, has demonstrated promise in a limited subset of tumors. Here, we target unexplored skull base tumors using a fast simultaneous two-channel stimulated Raman scattering (SRS) imaging technique and a new pseudo-hematoxylin and eosin (H&E) recoloring methodology. To quantitatively evaluate the efficacy of our approach, we use modularized assessment of diagnostic accuracy beyond cancer/non-cancer determination and neuropathologist confidence for SRH images contrasted to H&E-stained frozen and formalin-fixed paraffin-embedded (FFPE) tissue sections. Our results reveal that SRH is effective for establishing a diagnosis using fresh tissue in most cases with 87% accuracy relative to H&E-stained FFPE sections. Further analysis of discrepant case interpretation suggests that pseudo-H&E recoloring underutilizes the rich chemical information offered by SRS imaging, and an improved diagnosis can be achieved if full SRS information is used. In summary, our findings show that pseudo-H&E recolored SRS images in combination with lipid and protein chemical information can maximize the use of SRS during intraoperative pathologic consultation with implications for tissue preservation and augmented diagnostic utility.

## Introduction

The overarching purpose of intraoperative consultations for tumor surgeries is to obtain crucial information that informs surgical treatment. The tools currently employed include microscopic assessment of a submitted specimen through cytological preparations or cryosectioning and subsequent staining of the tissue by hematoxylin and eosin (H&E). The challenges of generating a diagnostically adequate H&E slide of cryosectioned tissue are organ-specific. In central nervous system specimens, the main challenge often results from limited sample size and general texture heterogeneity of the submitted tissue. In such cases, texture heterogeneity manifests itself as a freeze artefact - the diffuse or focal splitting of sections or cellular rarefaction resulting in loss of diagnostic information^1^. The presence of these artefacts in microscopic sections can result in misinterpretations and subsequent diagnostic pitfalls^2^.

In addition to the inherent challenges of cryosectioning, the location of the tumor can add complexity to management. One group of tumors that poses many challenges is skull base tumors which include meningiomas, pituitary adenomas, schwannomas, hemangiopericytomas, chordomas, various types of sarcomas, carcinomas, and metastases among other entities^3^. Skull base tumors face unique challenges due to their low occurrence, presence in deep locations, proximity to critical neurovascular structures, and extension beyond anatomic boundaries^4^. Accurate intraoperative tissue diagnosis is essential during skull base tumor surgery to maximize tumor removal, as a non-resected tumor can lead to recurrence, treatment failure, and overall poor outcome^5^. In particular, a rapid diagnosis during the removal of skull base tumors can help the surgeon to choose how aggressive the resection should be. In addition, in some patients, a rapid diagnosis can help to delineate the margins of tumor resection. Additionally, there is an increasing need to preserve tissue from small pathology specimens for downstream molecular ancillary testing, and an intraoperative technique aimed at preserving tissue would be preferable in these cases.

Many advanced optical imaging techniques have been developed to detect neoplastic cells and to provide diagnostic information with varying degrees of success, including optical coherence tomography^6,7^, confocal microscopy^8^, two-photon fluorescence combined with second harmonic generation^9–11^, Raman spectroscopy^12^, and coherent Raman scattering (CRS) microscopy^13^. Among the previous work, CRS stands out amongst other imaging techniques as it provides both morphological and chemical information at a submicron resolution without any staining or chemical labels^14^. Two CRS methods have been established: coherent anti-Stokes Raman scattering (CARS) and stimulated Raman scattering (SRS). Both CARS and SRS use two pulse lasers (pump and Stokes) to excite intrinsic vibrational motions of molecules coherently and have been shown to be able to provide molecular contrasts that can aid cancer diagnosis^13^.

In particular, it was recently demonstrated that SRS microscopy could provide H&E equivalent information for pathologists to determine cancer subtypes, thereby offering the potential for replacing frozen sectioning as an intraoperative diagnostic tool^15,16^. Specifically, it has been shown that a two-color SRS imaging approach targeting the C–H transition of lipid and protein (Raman transition at 2850 cm^−1^ and 2930 cm^−1^, respectively) allow direct visualization of cell nuclei. The resulting images can be recolored using linear dependence of the signal on lipid and protein content, allowing for a simple and very close simulation of H&E, i.e., stimulated Raman histology (SRH). Orringer *et al*. and Hollon *et al*. have tested the efficacy of SRS microscopy as a potential intraoperative H&E staining alternative for cases including glial and metastatic neoplasms with 92% accuracy of tumor subtype determination as assessed by neuropathologists^15,16^. Another recent study using the same SRH approach further validated the visual comparison of SRH images to H&E as applied to gastrointestinal tract^17^. Other studies used two-color SRS images for cancer/non-cancer identification in laryngeal squamous cell carcinoma^18^ and glioblastoma infiltration in the brain^19^. However, these studies did not provide pathologist evaluation of SRH or SRS two-color images for cancer subtype determination and thus are insufficient to validate SRS as an alternative to histology. Similar to SRS, CARS microscopy has also been explored for cancer diagnosis^20–22^ based on chemical contrasts. Compared to SRH, CARS is further complicated by the presence of a non-resonant background^23–26^, which prevents direct conversion of CARS images to H&E images. Adding additional chemical contrasts from second harmonic generation and two-photon fluorescence, it has been shown that multicolor images can be generated and provides useful diagnostic information^27^. However, whether pathologists can use these images to provide meaningful cancer diagnosis requires further investigation.

While a few studies have validated SRH in limited settings, it remains unclear whether SRH is generally applicable to a diverse set of tumor types encountered in daily pathology practice. Employing two Raman transitions targeting lipids and proteins to generate an H&E alternative works well for entities with higher cytoplasmic lipid content as can be expected in glial neoplasms. This is because the cytoplasmic lipid signals are a primary source of contrast to visualize cell nuclei. However, skull base tumors are inherently more complex because high protein and low lipid concentrations in the cytoplasm or the stromal matrix are common and can pose a challenge in visualizing nuclei. To explore the utility and limitations of SRH in complex tumors, we have set out to conduct a more dedicated investigation of SRH application to skull base tumors. Additionally, we take a different approach from previous studies by focusing on the user (i.e., pathologist) in addition to SRH validation. With only a few publications testing the accuracy of diagnosing histological subtypes^15,16^, we emphasize assessment of how SRH performs at every step of the typical diagnostic workflow. Moreover, unlike previous studies, instead of training pathologists with SRH images, diagnosis in this study is performed without prior training and thus provides an unbiased assessment of the diagnostic accuracy achievable in an intraoperative setting.

We used a simultaneous two-channel SRS imaging method we and others have developed to enable rapid SRH assessment^28,29^. Specifically, we performed simultaneous two-channel acquisition by using two 90° phase shifted Stokes pulses for orthogonal lock-in detection. This approach allows us to acquire protein and lipid SRS images rapidly for large pieces of tissue by eliminating wavelength switching and sequential acquisition. We image fresh resected tumor tissue collected prospectively during intraoperative consultation and validate the diagnostic capability of SRH through modularized review by board-certified neuropathologists. Our results reveal that SRH is effective in most cases with 87% accuracy relative to H&E stained formalin-fixed paraffin-embedded (FFPE) sections. Moreover, we find that in cases with limited lipid signal to provide the necessary contrast for nuclei visualization, rendering diagnosis is challenging when using strictly SRH, and it can be significantly improved with SRS chemical information.

## Methods

### Tissue collection and preparation

Sixteen subjects undergoing operations for skull base tumors were recruited with the approval of the University of Washington Institutional Review Board over the course of 12 months, and all research was performed in accordance with relevant guidelines and regulations. Subject eligibility was determined during preoperative evaluation by the neurosurgeon (L.N.S.) participating in this study, and written informed consent was obtained from each subject before surgery. At the time of standard intraoperative frozen section consultation, a fresh tissue sample measuring 0.3 × 0.2 × 0.1 cm on average was allocated for this study. Each piece of fresh and unlabeled tumor sample was placed on a well depression glass slide, moistened with saline and covered with a coverslip in preparation for SRS imaging. Following SRS imaging, each sample was placed in neutrally buffered formalin, processed, and embedded in paraffin for permanent sections. Samples were then sectioned and stained with H&E for standard histopathological analysis. All SRS images with corresponding FFPE sections stained with H&E of skull base tumors were reviewed by a board-certified neuropathologist (G.J-S.), who selected representative portions of SRS images for histological analysis. Slides of frozen sections or cytological preparations performed at the time of intraoperative consultation from all studied cases were also reviewed in this study in order to compare the quality of SRS images with standard intraoperative techniques and to determine the diagnostic utility of SRS microscopy.

### SRS microscopy

**Figure 1:**
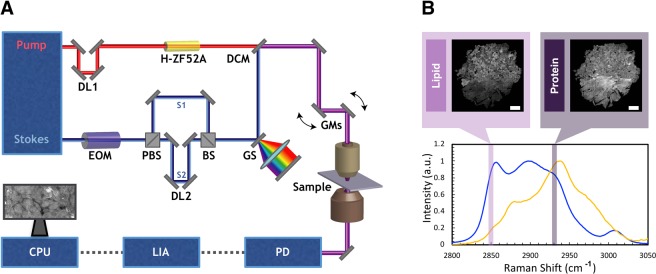
Experimental set up for SRS microscope and controls for lipid and protein channels. (**A**) The Stokes laser is modulated by an electro-optical modulator (EOM) at 20 MHz. Tunable laser is spectrally dispersed through glass rods (tunable) and Stokes laser is dispersed through a grating stretcher (GS). They are the spatially and temporally overlapped at a dichroic mirror (DCM) and directed onto a pair of galvanomirrors (GM). The beams are then sent through a laser scanning microscope with a 25x water immersion objective. The pump beam after the condenser is detected by a photodiode (PD). The signal is processed through lock-in amplifier (LIA). The images are collected using ScanImage on computer processing unit (CPU). (**B**) SRS spectra for oleic acid and bovine serum albumin (BSA) as controls for lipids and proteins, respectively.

Figure 1 A depicts simultaneous two-color SRS that was employed in our study^28,29^. Briefly, the Stokes and pump pulses provided by the fixed (1040 nm center wavelength, <200 fs, >1.5 W) and tunable (798 nm center wavelength, <150 fs, 1.0 W) outputs, respectively, of a dual-output oscillator (Insight DS+, SpectraPhysics) with a repetition rate of 80 MHz. The Stokes pulse was modulated at 20 MHz using an electro-optic modulator (EOM) and split into two separate arms, one of which was then delayed by 12.5 ns (1/80 MHz) relative to the other. The two arms were recombined using a 50:50 beamsplitter and stretched to 2 ps using a grating-based pulse stretcher^30,31^. The tunable source was centered at 798 nm, and the pulses were dispersed using 60 cm of glass rods. The average spectral width is estimated to be 300 cm^−1^ ^31^. The pump and Stokes beams were then combined with a dichroic mirror and overlapped temporally using a delay line in the tunable arm. The resulting beam was sent into a home-built laser scanning microscope. A 25X Olympus water immersion objective (NA = 1.05) was used to focus the beams onto the tissue sample. At the focus, the Stokes beams had an average power of 30 mW each, and the pump had an average power of 40 mW. After passing through the condenser, the Stokes beam was filtered out, and the pump reached a silicon photodiode. SRS signal was detected with a lock-in amplifier. The two orthogonal output of the lock-in amplifier provided the simultaneous two-channel SRS signals for pseudo H&E rendering^28^. Figure 1B depicts examples of SRS spectra for oleic acid and bovine serum albumin (BSA) as controls for lipids and proteins respectively.

### Image processing

**Figure 2:**
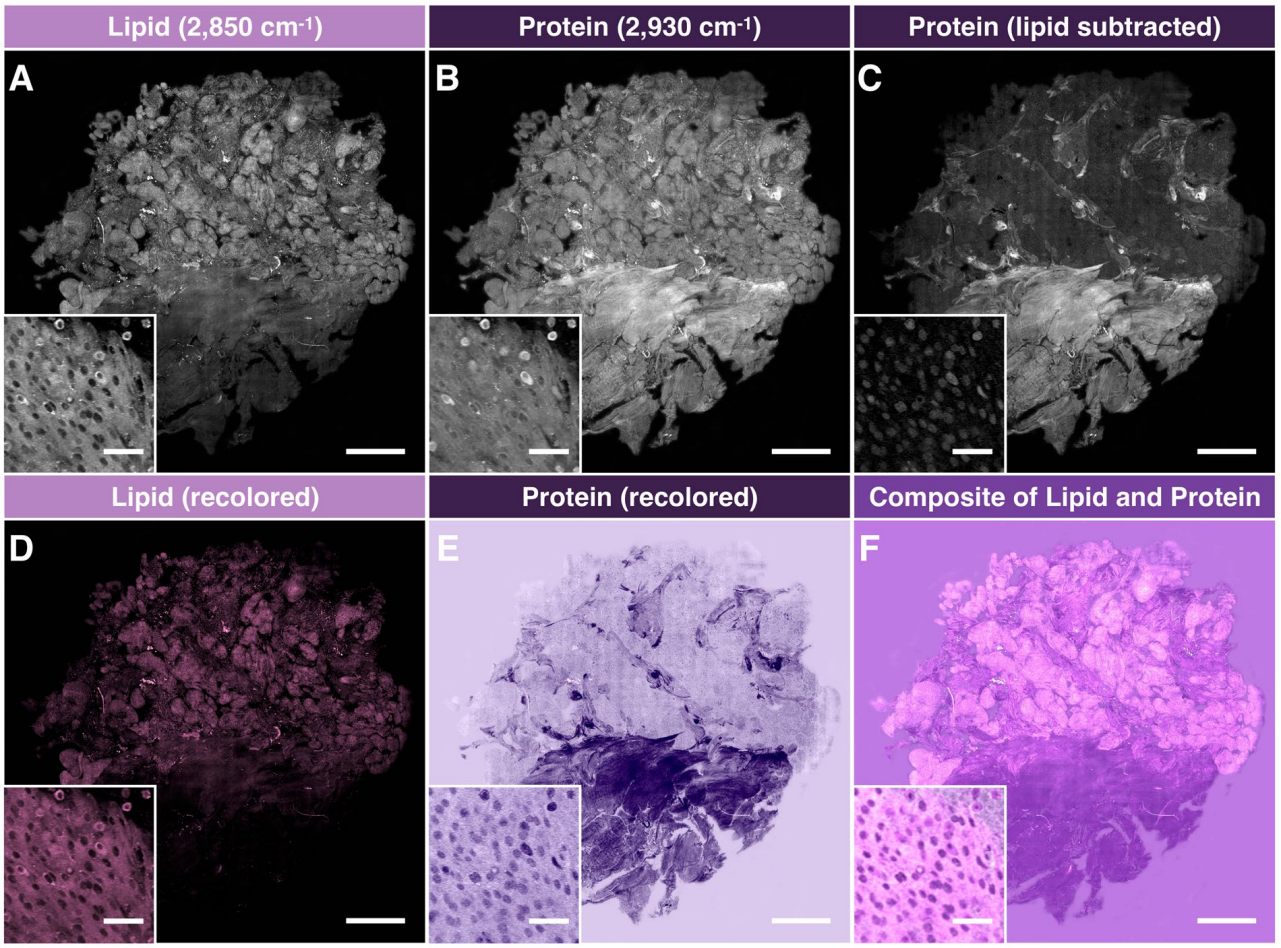
Image processing of stitched SRS imaging data (Meningioma, WHO grade I). (**A**) Stitched field-normalized data for lipid channel. (**B**) Stitched field-normalized data for protein channel. (**C**) Lipid data subtracted from protein, utilizing lipid and protein images in (**A,B**). (**D**) Recoloring result of lipid data. (**E**) Recoloring result of protein data. (**F**) Composite image of (**D,E**). Whole tissue scale bar: 1 mm. Inset scale bar: 50 *μ*m.

Figure 2 details how SRS images were processed into the pseudo-H&E images to be given to neuropathologists for analysis. The acquired lipid (Fig. 2A) and protein (Fig. 2B) images (285 *μ*m × 285 *μ*m per frame) were field-normalized and stitched to recover a complete tissue image (2–6 mm in size). Subsequently, the lipid channel was subtracted from protein channel, as previously demonstrated, to generate lipid corrected protein image (Fig. 2C)^32^. Using a custom-generated color scale in Image J, the lipid and lipid-corrected protein channels were recolored to match the conventional H&E (Fig. 2D,E) and combined to form a single image for histological analysis by pathologists (Fig. 2F).

### Survey methodology

A validation survey was conducted in order to establish the diagnostic utility of SRS simulated H&E images (SRH) compared to standard intraoperative pathology, which typically includes the frozen section and cytological preparation of the specimen routinely stained with H&E. The survey was performed by three neuropathologists (P.J.C., C.S.L., and L.F.G.-C.) who were blind to the diagnosis. They received a short clinical and radiographic summary of each case to mimic the circumstance of an intraoperative consultation but had no knowledge of SRS imaging and no training on SRH images. All sixteen studied cases were enrolled in the survey, which consisted of three phases: 1. Review of SRS generated images (SRH); 2. Review of the H&E-stained FFPE sections of the same specimen, and; 3. Review of the H&E-stained frozen sections or cytological preparations performed at the time of the neoplasm extraction for intraoperative diagnosis. Each phase included a series of multiple-choice questions focused on neoplastic appearance (epitheliod, spindle cell, myxoid, chondroid, or other), architectural pattern (lobular, fascicular, glandular, nested, papillary, sheeting, or other), and nuclear shape (rounded, elongated, and other). After the initial assessment, the evaluators participated in the diagnostic interpretation of each case that included differential diagnosis (a list of neoplasms that fit the neoplasm description, architectural pattern, and nuclear shape they have assigned to the case in combination with a clinical and radiographic summary provided) and final diagnosis. Finally, each participant was required to quantify their level of confidence in their diagnosis. The results of the survey were compared to the assessment by collaborating pathologist G.J.-S. (histopathological features and differential diagnosis) and the neuropathology report (final diagnosis).

### Statistical analysis of survey data

For each pathologist, percent agreement and Cohen’s kappa (*κ*_*C*_) were calculated while comparing each modality (SRH, H&E frozen, and H&E permanent) to the assessment by the collaborating pathologist G. J.-S. (histopathological features and differential diagnosis) and the neuropathology report (final diagnosis) for individual case using R software. Additionally, the relative accuracy as a ratio of SRH and H&E FFPE percent agreement was calculated for comparison of SRH to the golden standard of diagnosis. Fleiss’ kappa (*κ*_*F*_) was calculated to assess interpathologist reliability.

## Results and Discussion

### SRH images provide cytoarchitectural visualization necessary for diagnosis

**Figure 3:**
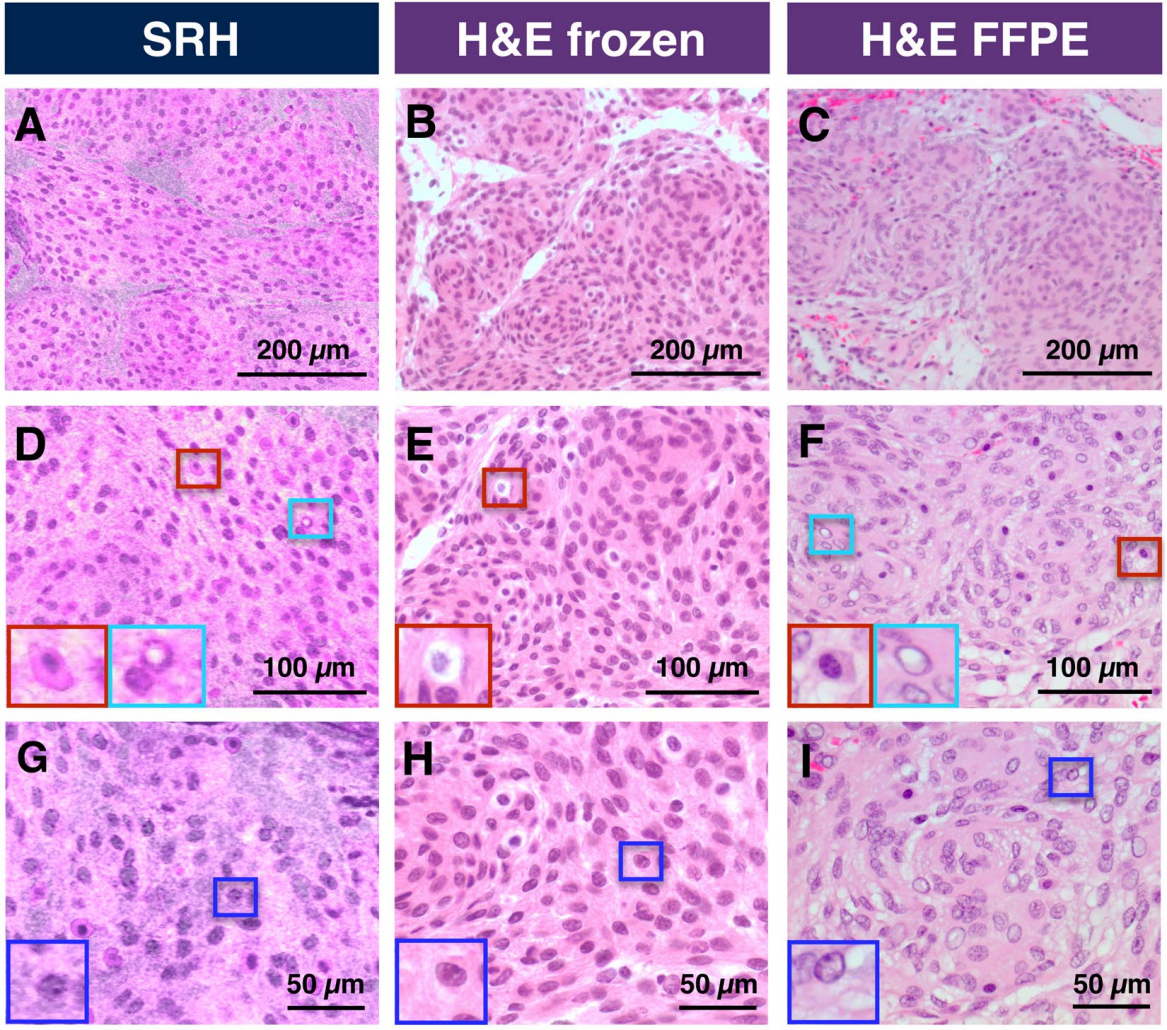
Comparison of cellular features available with SRH *versus* conventional H&E stained slides in representative case of Meningioma WHO grade I. (**A–C**) Comparison of architecture features. (**D–F**) Increased magnification highlighting macrophages (red box) verified by immunohistochemistry and intranuclear inclusions (cyan box). (**G–I**) Nuclear features with easily identifiable nucleolus (blue box).

For a pathologist, architectural and cytological features are necessary for the diagnostic process. We use a studied meningioma case in Fig. 3 as an example of cytoarchitectural visualization available with SRH images. The characteristic lobular architectural growth pattern of epithelioid cells is as apparent in SRS images as it would be at a lower magnification of H&E stained frozen or FFPE sections (Fig. 3A–C). At high resolution, the SRH image shows clear cytomorphology such as round to oval nuclei with bland chromatin and inconspicuous nucleoli (Fig. 3G–I). Moreover, scattered intranuclear pseudoinclusions, common histological features identified in meningiomas, are also visualized on SRH (Fig. 3D). Identical histological features are present in concurrent frozen and concomitant FFPE sections (Fig. 3H,I).

Furthermore, sufficient cytomorphological features can be identified to determine other cell types. Figure 3D–F show lipid-rich cells identified and marked by a red box as macrophages (confirmed by immunohistochemistry). While in the case of meningioma, these cells are not considered diagnostically essential, in the case of breast cancer, tumor-associated macrophages have been explored as a prognostic marker^33,34^.

### SRH images reveal diagnostic features in a broad range of skull base tumors

**Figure 4:**
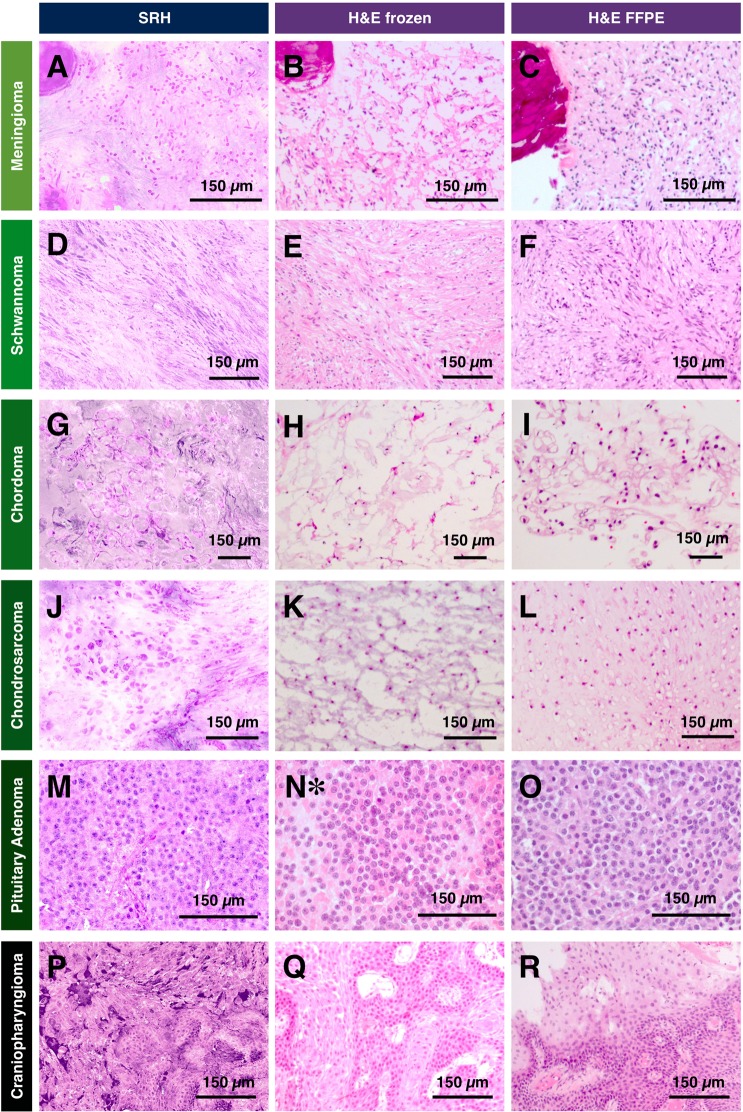
Comparison of SRH with conventional histological preparations of skull base tumors. (**A–C**) Meningioma, WHO grade I. (**D–F**) Schwannoma, WHO grade I. (**G–I**) Chordoma. (**J–L**) Chondrosarcoma, grade 2. (M,N*,O) Sparsely granulated somatotroph adenoma. (**P–R**) Papillary craniopharyngioma (BRAF-mutant). *The case warranted cytological preparations only during intraoperative consultation.

An accurate pathologist interpretation of unique histopathological features is essential during a neurosurgical procedure. For SRH to be applicable in an intraoperative setting, SRH must deliver clear and easy to interpret images with sufficient information. To examine the capability of SRH at providing useful histopathological information, we image a broad range of skull base tumors including nine meningiomas, three schwannomas, one chordoma, one chondrosarcoma, one pituitary adenoma, and one papillary craniopharyngioma. Figure 4 highlights typical diagnostic features of selected skull base tumors captured by SRH in comparison with H&E stained frozen sections of the same case and H&E stained FFPE sections of the same tissue.

The advantage of SRH is the ability to image the sample without sectioning, which eliminates challenges introduced by freeze artefact from cryosectioning. This issue is exacerbated when tissue samples are challenging to cut due to the heterogeneity of texture. The three cases that serve as an example (meningioma, chordoma, and chondrosarcoma) are shown Fig. 4. Figure 4A,G,J demonstrate well-preserved diagnostic features in SRH images for meningioma, chordoma, and chondrosarcoma, respectively. In contrast, Fig. 4B,H,K show corresponding H&E stained frozen sections distorted by freeze artefact. FFPE sections providing a reference for the typical appearance of histological features are shown in Fig. 4C,I,L.

In the case of meningioma, the texture heterogeneity is caused by psammoma bodies (round collection of calcified material). This is a good example of the challenges faced when calcified material is present during cryosectioning. Psammoma bodies are diagnostically helpful when suspecting meningioma, which mitigates poor tissue preservation in this case specifically. On the other hand, conserving features in case of chordoma and chondrosarcoma is important for accurate identification. Figure 4G,I show chordoma with vague small clusters and individual epithelioid cells with pale vacuolated cytoplasm (physaliphorous cells) and prominent dense extracellular protein-rich myxoid matrix that are missing in frozen section images. Similarly, one studied chondrosarcoma (low-grade per neuropathology report) demonstrated scattered mononucleated neoplastic cells in lacunae found in an abundant cartilaginous protein-rich background (Fig. 4J,L). These three examples highlight SRH advantage over the conventional frozen section in preventing loss of cytoarchitecture due to the extensive freeze artefact and tissue texture heterogeneity. When freeze artefacts are not a significant issue, the diagnosis on frozen section analysis can still be challenging due to ambiguous histomorphology in spindle neoplasms including schwannoma (Fig. 4D–F) and meningioma without psammoma bodies (Fig. 3). With SRH, the different recoloring scheme can be used to highlight those features as will be discussed later. Additionally, because SRH is non-destructive, any imaged tissue can be saved for downstream histopathological analysis, and it is a significant advantage in cases where there is limited tissue.

In other studied types of tumors, similar morphological features found typically in FFPE sections are sufficiently replicated in SRH. For example, SRH of studied pituitary adenoma reveals sheets of relatively monotonous neuroendocrine cells with a moderate amount of cytoplasm and rounded nuclei with bland and occasionally stippled chromatin, depicted in Fig. 4M. These histological features agree well with those observed in a concomitant H&E-stained FFPE section (Fig. 4O) as well as concurrent cytological preparation (Fig. 4N). Additionally, our study includes a craniopharyngioma, a squamous epithelial neoplasm characterized by cauliflower-like papillary structures and fibrovascular core, which are visible on SRH image (Fig. 4P), FFPE (Fig. 4R), and corresponding frozen sections stained with H&E (Fig. 4Q).

Finally, SRH provides information on chromatin appearance, which can be used as evidence of preoperative treatment such as embolization. Preoperative endovascular embolization, adjunctive treatment of meningioma, was performed in 5 out of 9 studied meningiomas, and in one studied case resulted in noticeable cytomorphological changes captured on SRH (Supplementary Fig. 1), including the vague architectural structure and neoplastic cells with pyknotic nuclei (Fig. 4A). These histopathological features were also identified on both conventional H&E-stained FFPE sections of the same specimen and corresponding frozen sections (Fig. 4B,C). To contrast, a meningioma case without prior embolization discussed earlier can be referenced (Fig. 3 and Supplementary Fig. 1).

Qualitative comparison of SRH images to H&E stained frozen sections shows that SRH offers an advantage over frozen section by avoiding diagnostic pitfalls due to freeze artefact. The pseudo-H&E recolored SRS images offer comparable diagnostic features that are available in H&E stained FFPE with the advantage of being non-destructive and preserving tissue for downstream diagnostic tests.

### Diagnostic accuracy of SRH images compared to conventional H&E based techniques

In order to establish the diagnostic utility of SRH, we administer a survey which assesses the interaction of a given neuropathologist with SRH, H&E stained frozen, and FFPE section slides. Unlike previous studies^15,16^, our survey included modularized assessment of the pathologist experience with SRH testing beyond final diagnostic accuracy. Typically, a pathologist uses histopathological features including neoplasm description, architectural pattern, and nuclear shape, among others in combination with clinical history and neoplasm location to progress from differential diagnosis to the final diagnosis. Because the generation of the final diagnosis is involved, we benefit from understanding what histopathological features were discrepant on SRH. Moreover, we are comparing how H&E stained frozen, and FFPE sections perform in the same situation as SRH to isolate quality drawbacks of SRH from possible pathologist variability inherent to the diagnostic process. Table 1 shows a summary of our survey findings. The percent agreement highlights the absolute proportion of cases that agree with the collaborating pathologist G. J.-S. (histopathological features and differential diagnosis) and the neuropathology report (final diagnosis). Concordance based on Cohen’s kappa (*κ*_*C*_), is also reported. Interpathologist reliability is assessed using Fleiss’s kappa (*κ*_*F*_). To limit the impact of interpathologist variation, we report a relative accuracy defined as a ratio of SRH percent agreement and H&E FFPE to highlight the performance of SRH relative to the gold standard.

**Table 1:** Survey results, comparing SRH *versus* conventional tissue processing and staining.

	Neuropathologist 1			Neuropathologist 2			Neuropathologist 3			Interpathologist reliability (*κ*_*F*_)		
	SRH	H&E frozen	H&E FFPE	SRH	H&E frozen	H&E FFPE	SRH	H&E frozen	H&E FFPE	SRH	H&E frozen	H&E FFPE
**Neoplasm Description:**												
Percent Agreement	88%	88%	88%	75%	88%	81%	75%	75%	75%			
Concordance (*κ*_*c*_)	0.78	0.80	0.80	0.52	0.78	0.66	0.61	0.56	0.59	0.48	0.78	0.63
Relative Accuracy	**100%**	—	—	**92%**	—	—	**100%**	—	—			
**Architectural Pattern:**												
Percent Agreement	75%	75%	75%	69%	88%	81%	44%	56%	50%			
Concordance (*κ*_*c*_)	0.64	0.65	0.65	0.58	0.83	0.74	0.34	0.48	0.43	0.28	0.38	0.41
Relative Accuracy	**100%**	—	—	**85%**	—	—	**88%**	—	—			
**Nuclear Shape:**												
Percent Agreement	94%	100%	94%	94%	94%	94%	88%	94%	94%			
Concordance (*κ*_*c*_)	0.87	1.00	0.86	0.86	0.86	0.86	0.73	0.86	0.86	0.64	0.91	0.82
Relative Accuracy	**100%**	—	—	**100%**	—	—	**93%**	—	—			
**Differential:**												
Percent Agreement	94%	100%	100%	94%	100%	100%	94%	100%	100%			
Concordance (*κ*_*c*_)	0.90	1.00	1.00	0.90	1.00	1.00	0.90	1.00	1.00	—	—	—
Relative Accuracy	**94%**	—	—	**94%**	—	—	**94%**	—	—			
**Final Diagnosis:**												
Percent Agreement	88%	94%	100%	75%	94%	81%	81%	100%	94%			
Concordance (*κ*_*c*_)	0.80	0.90	1.00	0.61	0.90	0.68	0.66	1.00	0.90	0.57	0.87	0.79
Relative Accuracy	**88%**	—	—	**85%**	—	—	**87%**	—	—			
**Confidence score**	***3.13***	***3.50***	***3.50***	***2.31***	***2.81***	***3.31***	***2.94***	***3.63***	***3.56***			

Relative accuracy is calculated as ratio of SRH percent agreement and H&E FFPE. Confidence score is assigned as follows: 4 - highly confident, 3 - confident, 2 - somewhat confident, 1 - not at all confident.

Overall, the SRH percent agreement for histopathological features is approximately the same as the percent agreement for FFPE and frozen sections between neuropathologists. However, the interpathologist reliability for SRH is consistently lower within a given histopathological feature. These findings could be explained by the fundamental difference of tissue handling in SRH as measurements are conducted on fresh tissue and the final appearance of cellular features as well as tissue architecture could be slightly different when compared to frozen and FFPE. Additionally, the inherent differences in color scale between SRH and H&E stained sections could affect the assessment of the architectural growth pattern. For example, the myxoid and chondroid architectural growth patterns are not visualized sufficiently, as supported by our survey, on SRH due to the proteinaceous background being recolored darker than what is found typically on H&E. Despite the possible shortcomings of SRH when evaluating individual histopathological features, the differential diagnosis was developed successfully (average of 94%, Table 1).

**Figure 5:**
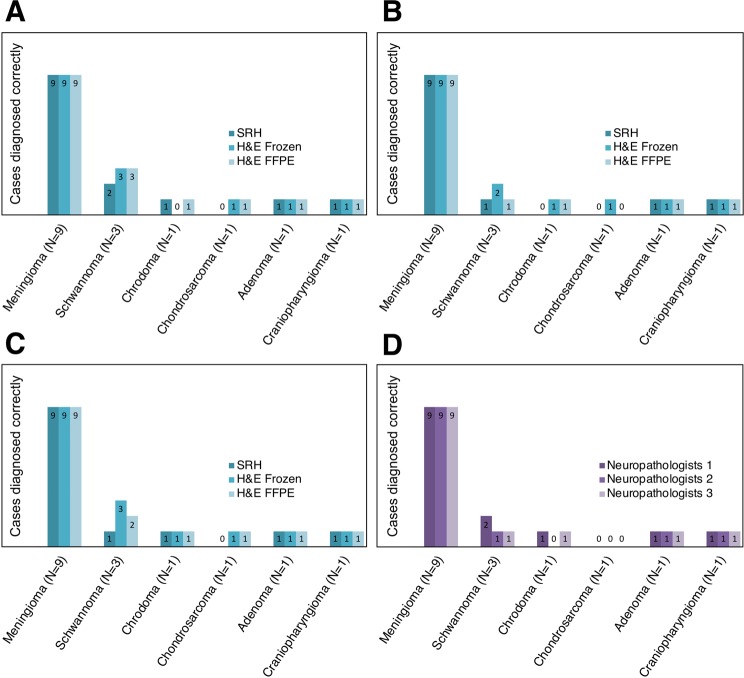
Depiction of correctly diagnosed cases for each neuropathologist and modality. (**A–C**) Neuropathologist 1, 2, and 3, respectively. (**D**) SRH only comparison for all neuropathologists.

Using the histopathological features and clinical history, as mentioned above, the neuropathologist narrowed down the differential diagnosis to the final diagnosis with an average percent agreement of 81% (Table 1). Figure 5 shows a closer look at the cases used in the assessment and specifically what cases received the discrepant diagnoses. Based on our evaluation, we find two tumor types that are most prone to discrepancies in the final diagnosis when using SRH images: schwannoma and chondrosarcoma (Fig. 5D). In the case of chondrosarcoma, only one of the three neuropathologists includes the entity on their differential diagnosis. One plausible cause for this is the component composition of this tumor type. Chondroid neoplastic cells produce a cartilaginous matrix low in lipid content with occasional intracytoplasmic hyaline globules in lower grade tumors. Because both the cartilaginous matrix and hyaline globules will have SRS signal in the protein channel with minimal lipid content for additional contrast, chondrosarcoma cases can be challenging to diagnose using SRH (Fig. 4J). Such an issue is mainly due to the requirement of having to match the H&E color scheme.

For the case of schwannomas, we have found that neuropathologists frequently qualify their decision when arriving at a final diagnosis for spindle cell tumors such as meningioma and schwannoma. For all neuropathologists, the differential diagnosis for such cases is correct. Pathologists may remain cautious with final diagnoses during intraoperative consultations, explaining that spindle cell neoplasm can be either schwannoma or meningioma, with the understanding that differentiation between those two entities will not have a significant impact on the immediate surgical management. In such cases, the final classification can be deferred to FFPE sections where additional histological examination and ancillary stains performed as needed can provide the necessary information to arrive at a definitive diagnosis.

Finally, SRH provides enough information for a neuropathologist to render the final diagnosis with an average percent agreement of 81% and relative accuracy of 87% (Table 1). The neuropathologist rated confidence score correlates with percent agreement. This correlation is expected as the more confident a neuropathologist feels using SRH images, the more accurate their diagnosis is expected to be. Although the objective of our study was to see how SRH performs without the use of training data sets, it is relevant to highlight that slight differences between SRH and conventional H&E staining impacts confidence and, by extension, diagnostic accuracy. This issue could be mitigated with SRH interpretation training that would be necessary for clinical application of SRH.

### Additional chemical information improves diagnosis of SRH

After evaluating the types of tumors that are discrepant on SRH images, we identified that low stromal lipid concentration (as in the case of chondrosarcoma) could be challenging for pseudo-H&E recoloring. In conventional H&E staining, hematoxylin preferentially stains nucleic acids dark purple, highlighting nuclear features, whereas eosin stains protein content various shades of pink and red. Pseudo recoloring used in SRH relies on contrast from lipid and protein to recolor protein signal purple, enabling clear visualization of the nucleus. However, the requirement to pseudo-H&E recolor SRS images in SRH limits how chemical information from SRS is presented, which underutilizes the differences in protein and lipid in stromal or cytoplasmic components. The information on lipids is not available in conventional H&E.

Although this study is focused on evaluating the diagnostic utility of SRH, it is important to emphasize that protein and lipid information from SRS is available to augment the use of SRH in intraoperative consultation. This can be exemplified in two different cases.

**Figure 6:**
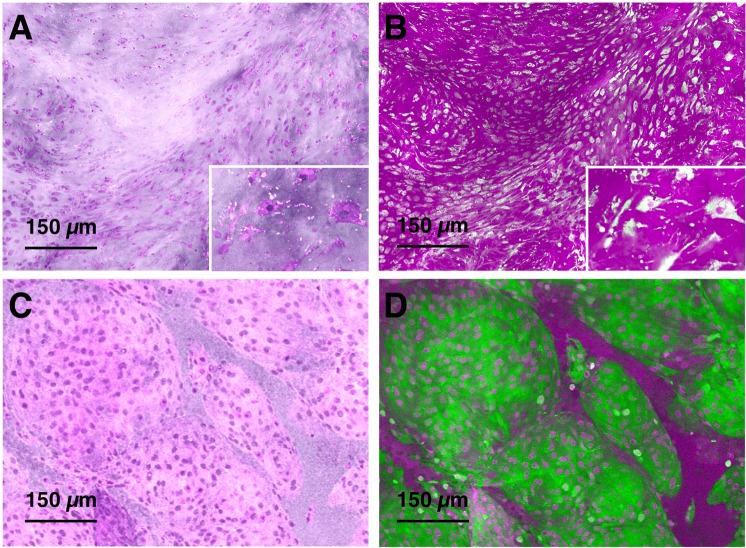
Chondrosarcoma *versus* meningioma. (**A**) Pseudo-H&E recolored SRH of chondrosarcoma case. (**B**) Lipid (green) and protein (magenta) SRS of chondrosarcoma case. (**C**) Pseudo-H&E recolored SRH of meningioma case. (**D**) Lipid (green) and protein (magenta) SRS of meningioma case.

Firstly, the studied chondrosarcoma case was misclassified as meningioma due to perceived similarities on SRH (Fig. 6A,C). However, direct analysis of the protein and lipid SRS images of SRS recolored in magenta and green (Fig. 6D *versus* 6B) shows a high lipid signal in the case of meningioma, while in chondrosarcoma, the supporting cartilaginous matrix is protein-rich. The SRS-based data matches the general knowledge that chondrosarcoma cells can be found in a matrix that is predominantly cartilage, made almost exclusively of protein. Information provided by SRS can thus facilitate a confident diagnosis of chondrosarcoma.

**Figure 7:**
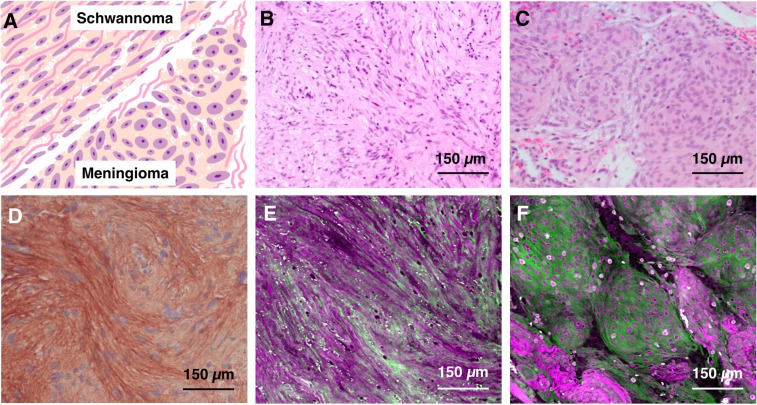
SRS images improve the identification of collagen. (**A**) Depiction of architectural and morphological differences between meningioma and schwannoma with particular focus on collagen fibers. (**B**) H&E of schwannoma. (**C**) H&E of meningioma. Protein signal is shown in magenta while lipid signal is shown as green. (**D**) Sample of collagen type IV antibody stained schwannoma case. (**E**) SRS based image of schwannoma. (**F**) SRS based image of meningioma.

Secondly, the occasional challenge of differentiating meningioma and schwannoma cases can be overcome with SRS lipid and protein information without having to resort to immunohistochemistry staining. In schwannoma, the collagen fibers often are present between neoplastic cells in contrast to meningioma, where the collagen fibers usually surround the group of neoplastic cells that can be found in a lobular pattern (Fig. 7A). These histological features are subtle when using H&E-stained FFPE sections (Fig. 7B,C), but can be distinguished with immunohistochemistry (Fig. 7D). To assist with the differentiation of meningioma from schwannoma using SRS, we can highlight collagen fibers using magenta (protein) and green (lipid) colors in combination with simple linear remapping that correlates the color intensity to the concentration of the constituent of interest. In addition to simulated H&E, the protein and lipid data contrasted in the new color scheme allows for more efficient highlighting of collagen fibers and determining whether they are present in between neoplastic cells (Fig. 7E) or are present around meningioma lobules (Fig. 7F), allowing us to separate schwannoma from meningioma.

In summary, we highlight in Figs. 6 and 7 that using the lipid and protein chemical information, a pathologist could better visualize the collagen fibers in schwannoma cases and discern collagenous matrix of chondrosarcoma from lipid-rich tumors such as meningioma respectively. Furthermore, the SRS ability to detect collagen fibers can be used as a surrogate for immunohistochemistry, which could improve the diagnostic process intraoperatively in addition to saving money on expensive antibody reagents.

### Conclusion and future outlook

SRH has shown great promise in intraoperative diagnosis of a limited subset of neoplastic entities, particularly glial tumors. However, the broad utility of SRH and challenges it may face in more diverse tissue remain largely unknown. Furthermore, the evaluation of the diagnostic accuracy conducted in previous studies has only focused on comparing SRH to H&E frozen sections and clinical diagnosis rendered intraoperatively^15,16^. However, the gold standard in surgical pathology is the use of H&E stained FFPE sections in combination with ancillary studies, including immunohistochemistry, which requires time-consuming tissue processing as well as pathological interpretation. We address the gaps in testing SRH diagnostic capability using the reported original neuropathological diagnosis established for a given case and compare SRH to conventional modalities while accounting for interpathologist variability. In order to thoroughly assess the diagnostic utility of SRH, it is important to modularize the evaluation process to determine precisely the areas where SRH based technology needs improvement. We achieve that by assessing the efficacy at multiple steps on the way to final diagnosis as well as checking the neuropathologist confidence when using the new modality.

Taking our unique evaluation approach, we find that neuropathologists were able to establish a neoplasm description, architectural pattern, and nuclear shape with practically the same percent agreement as conventional modalities. Following the diagnostic process, we find that neuropathologists generate differential diagnosis successfully and final SRH diagnostic capability is very close to conventional modalities. In addition to demonstrating the diagnostic capabilities of SRH on this subset of tumors, we demonstrate supporting findings that SRS chemical information with lipid and protein can further help diagnostic process and possibly reduce the need of immunohistochemistry use in selected cases.

At the same time, one of the main challenges that the SRS technique is likely to face in broader organ applications is the reliance on lipid and protein contrast to visualize nuclear features which are important to render a pathological diagnosis. For many neoplastic entities, protein/lipid-based simulation of H&E works well. However, for situations where lipid is limited in the cytoplasm, or otherwise nearby stroma, a map of local protein concentration provided by SRS might not be sufficient for successful visualization of a nucleus and general cellular morphology. New recoloring schemes can be considered to mediate such a problem. Nuclear segmentation has been reported in H&E stained slides, fluorescence images, and SRS images^35–37^. With nuclear segmentation, a separate recoloring technique can be employed. However, incorporating separate recoloring techniques must be generalized across different entities to reduce user variability and avoid inconsistent simulation of H&E. Such inconsistencies can prompt the pathologist to misclassify the type of neoplasm with a detrimental consequence to patients. Moreover, when designing new recoloring schemes, it is essential to use a consistent method without *a priori* knowledge of H&E as such knowledge would not be available in an intraoperative setting.

In conclusion, we have studied a diverse set of skull base tumors using fast simultaneous 2-channel SRS imaging and new pseudo-hematoxylin and eosin (H&E) recoloring methodology. Due to label-free non-destructive features of SRS technique, we demonstrated that most challenges with cryosectioning and limited amounts of fragile tissue during intraoperative consultation could quickly be addressed using SRS based approach. Using a diverse set of tumors, we determined the potential drawbacks of pseudo-H&E recoloring in selected cases involving insufficient nucleus visualization. While agreeing with previous work on SRS accuracy in intraoperative setting, we determined that SRS is capable of mostly matching the conventional H&E based technique and more importantly, providing additional diagnostically useful information. By following the diagnostic process that a pathologist uses, we discovered that pseudo-H&E recoloring, lipid/protein chemical information, and additional pathologist training to interpret this new information must be considered in tandem to bring SRS into clinical practice.

## Supplementary information

Supplementary information.
