# Supplementary material for: Intraoperative assessment of skull base tumors using stimulated Raman scattering microscopy
Source: Sci Rep. 2019 Dec 31;9:20392. doi: 10.1038/s41598-019-56932-8 (PMC6938502; doi:10.1038/s41598-019-56932-8)
Supplement: Supplementary file 1 — Supplementary information. [file 41598_2019_56932_MOESM1_ESM.pdf]

Intraoperative assessment of skull base tumors using stimulated Raman scattering microscopy

Kseniya S. Shin[1,2], Andrew T. Francis[1], Andrew H. Hill[1], Mint Laohajaratsang[1], Patrick J. Cimino[3], Caitlin S. Latimer[3], Louis F. Gonzalez-Cuyar[3], Laligam N. Sekhar[4], Gordana Juric-Sekhar[3,4,*], and Dan Fu[1,]**

[1]Department of Chemistry, University of Washington, Seattle, 98125-1700, USA
[2]School of Medicine, University of Washington, Seattle, 98125-1700, USA
[3]Department of Pathology, University of Washington, Seattle, 98125-1700, USA
[4]Department of Neurological Surgery, University of Washington, Seattle, 98125-1700, USA
[*]gordana@uw.edu
[**]danfu@uw.edu

Supplementary information

Case No	Age/Sex	Symptoms	Location of the neoplasm	Neuropathologic diagnosis
1	62/M	diplopia, third nerve palsy	L clinoid process and adjacent cavernous sinus	Meningioma, WHO grade I
2	64/F	dizziness, nausea	L posterior fossa	Meningioma, WHO grade I
3	39/F	headache, uncontrollable eye movements	L petrous apex	Chondrosarcoma, grade 2
4	62/F	altered mental status	R planum shenoidale	Meningioma, WHO grade I
5	45/M	headache, ataxia	R cerebellopontine angle cystern and internal auditory canal	Cellular schwannoma, WHO grade I
6	46/F	Bil deteriorated vision and R CN VI palsy	medial sphenoid wing	Schwannoma, WHO grade I
7	34/F	headache	R temporobasal near petrous bone	Meningioma, WHO grade I
8	54/F	incidental finding	midline clivus	Chordoma
9	46/F	headache	L torcular/posterior fossa/skull base	Meningioma, WHO grade I
10	71/F	R deteriorated vision	planum sphenoidale	Meningioma, WHO grade I
11	58/F	R hypoacusia	R cerebellopontine angle	Schwannoma, WHO grade I
12	54/F	L visual disturbance	L spheno-orbital wing	Meningioma, WHO grade I
13	33/M	acromegaly	sellar and suprasellar	Sparsely granulated somatotroph adenoma
14	60/M	headache, hyposmia, L hypacusia	planum shenoidale	Meningioma, WHO grade I
15	49/M	bitemporal hemianopsia	3rd ventricle/hypothalamic area	Papillary craniopharyngioma (BRAF-mutant)
16	45/F	L visual disturbance	L cavernous sinus, middle cranial fossa and sella	Meningioma, WHO grade I

Supplementary Table 1. Descriptive case statistics. Bil-bilateral. F-female. M-male. WHO-world health organization

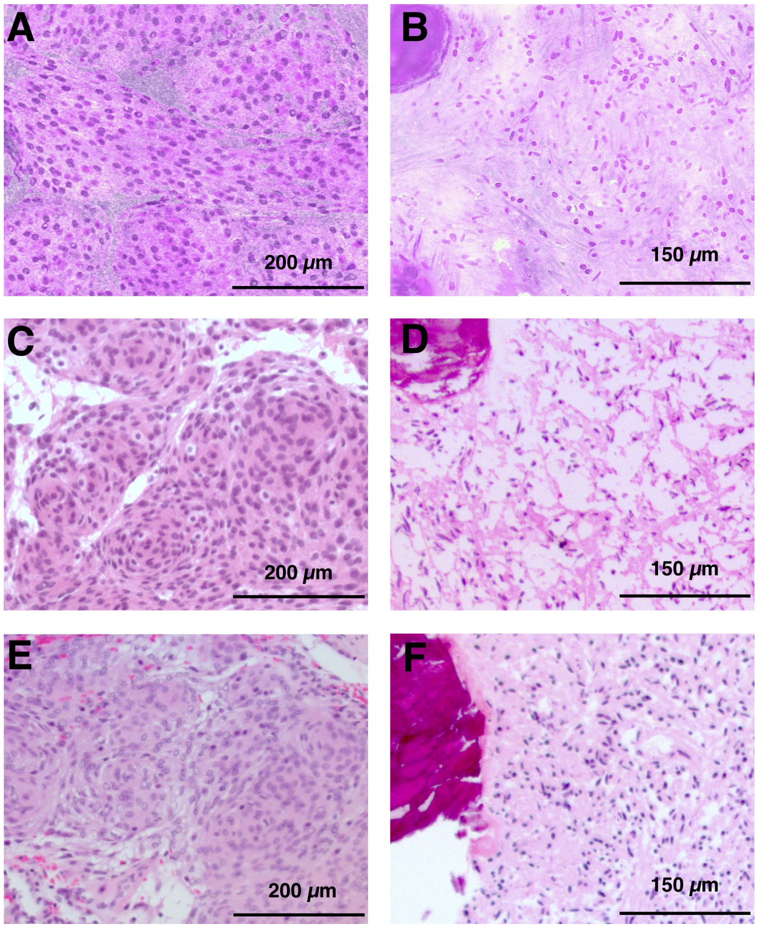

Supplementary Figure 1. Comparison of meningioma with and without preoperative endovascular embolization treatment. A) SRH of meningioma case without preoperative embolization. B) SRH of meningioma case with preoperative embolization. C) H&E stained frozen section of meningioma case without preoperative embolization. D) H&E stained frozen section of meningioma case with preoperative embolization. E) H&E stained FFPE section of meningioma case without preoperative embolization. F) H&E stained FFPE section of meningioma case with preoperative embolization.
